# Congenital Tuberculous Lymphadenitis in a Preterm Neonate Born to a Comatose Mother in a High TB‐Burden Setting: A Case Report

**DOI:** 10.1155/crpe/9912027

**Published:** 2025-12-26

**Authors:** Jason Nzanzu Kikuhe, Eugnénie Mbindule Kalere, Castram Mbusa Makata, Josias Kasereka Songya

**Affiliations:** ^1^ Department of Surgery, University of Goma, Goma, Democratic Republic of the Congo; ^2^ Platinum Head and Neck Centre Goma, Goma, Democratic Republic of the Congo; ^3^ ISETEM (Virunga Higher Institute of Medical Techniques), Goma, Democratic Republic of the Congo; ^4^ Department of Pediatrics, Great Lakes Free University, Goma, Democratic Republic of the Congo

**Keywords:** antenatal screening, congenital tuberculosis, maternal coma, preterm neonate, resource-limited settings, vertical transmission

## Abstract

**Background:**

Congenital tuberculosis (TB) is a rare but often fatal condition, particularly in preterm neonates within high‐burden regions like the Democratic Republic of Congo (DRC). Diagnosis is complicated by nonspecific symptoms and resource limitations, with maternal critical illness further delaying detection and increasing vertical transmission risk.

**Case Presentation:**

This case report details a preterm male infant (30 weeks’ gestation) born vaginally to an HIV‐negative mother who experienced a 2‐month coma during pregnancy in the DRC. The neonate presented at 3 weeks with cervical lymphadenopathy, respiratory distress, and failure to thrive. Initial sepsis treatment with antibiotics failed. Postpartum maternal sputum testing confirmed pulmonary TB. Neonatal lymph node biopsy revealed caseating granulomas, and GeneXpert MTB/RIF confirmed *Mycobacterium tuberculosis* infection. The infant received weight‐adjusted antitubercular therapy (rifampicin, isoniazid, and pyrazinamide), leading to progressive clinical improvement.

**Conclusion:**

This case underscores congenital TB as a critical differential diagnosis in preterm infants with lymphadenopathy born to critically ill mothers in TB‐endemic areas. Maternal coma—a likely indicator of disseminated TB—masked symptoms, delayed diagnosis, and amplified transmission risk. The report advocates for integrating routine TB screening into antenatal care protocols for all critically ill pregnant women in high‐burden settings, irrespective of overt respiratory symptoms. Early maternal diagnosis and prompt neonatal treatment are essential to reduce mortality in this vulnerable population. Enhanced screening in high‐risk pregnancies represents an urgent public health priority for mitigating vertical TB transmission in resource‐limited contexts.

## 1. Introduction

### 1.1. Background

Tuberculosis (TB) remains a leading cause of morbidity and mortality worldwide, with sub‐Saharan Africa bearing 25% of the global burden. The Democratic Republic of Congo (DRC), a high–TB‐burden country, reports over 200,000 annual cases, yet congenital TB, a rare manifestation of vertical transmission, is frequently underdiagnosed due to nonspecific neonatal presentations and limited diagnostic capacity in resource‐constrained settings [1–3]. Congenital TB, defined as infection acquired in utero or during delivery, accounts for < 2% of pediatric TB cases and carries high mortality (up to 50%) if untreated [4]. Transmission occurs via hematogenous spread through the umbilical vein, aspiration of infected amniotic fluid, or direct contact during birth [5]. Preterm neonates are particularly vulnerable due to immature immune systems and overlapping clinical features with sepsis or congenital pneumonia [6]. Despite these risks, maternal TB is often undiagnosed antenatally, especially in critically ill or comatose women, further amplifying transmission risks [7]. This report presents a rare case of congenital tuberculous lymphadenitis in a preterm neonate born to a mother who experienced a 2‐month coma during pregnancy in the DRC. Maternal coma, a poorly described risk factor for vertical TB transmission, delayed both maternal diagnosis and neonatal intervention. By contextualizing this case within the challenges of TB‐endemic settings, we aim to highlight the diagnostic complexities of congenital TB in preterm neonates, emphasize the role of maternal critical illness in perpetuating transmission, and advocate for improved antenatal TB screening protocols in high‐burden regions, even among mothers with nonrespiratory presentations.

### 1.2. Case Presentation

Maternal history: In 2022, a woman aged 30 years from the DRC presented with a preterm pregnancy complicated by a 2‐month coma of unknown etiology during her third trimester. She had no prior history of TB or antenatal TB screening. The coma resolved spontaneously, but she remained bedbound with no further neurologic evaluation due to limited healthcare access. She delivered vaginally at 30 weeks of gestation without obstetric complications. Postpartum, she developed a persistent cough; sputum testing confirmed pulmonary TB (acid‐fast bacilli [AFB]) smear‐positive at 1‐week postpartum. She tested negative for HIV. It should be noted that we did not have access to the mother’s records or the results of all her tests performed at her hospital, as she was still hospitalized in a rural facility located 20 km from our specialized center. The brief history mentioned here was included in the referral note completed after interviewing the child’s caregiver (paternal aunt of the child). Neonatal presentation: A male infant was born weighing 1200 g with Apgar scores of 6 and 8 at 1 and 5 min, respectively. He required minimal resuscitation and was transferred to neonatal intensive care for prematurity management. At 3 weeks of life, he developed.

### 1.3. Submandibular Lymphadenopathy

**Figure 1:**
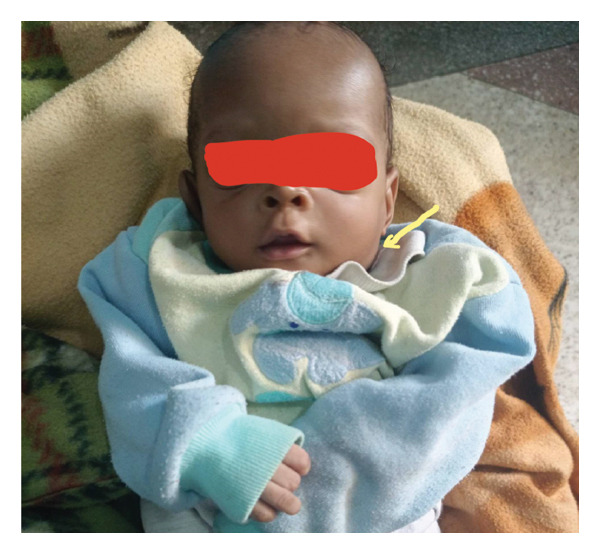
Submandibular lymphadenopathy.

Firm, nontender bilateral neck masses (2‐3 cm) without overlying erythema is shown in Figure 1.

The patient was treated in the pediatric ward for sepsis, receiving a 12‐day (5 weeks of age) course of antibiotics (ceftriaxone IV) with no clinical improvement. Two weeks later (7 weeks of age), the lymphadenopathy fistulized near the pinna of the ear, prompting referral to our ENT clinic for further evaluation of possible otitis media or mastoiditis. Examination of the ear canal and tympanic membrane revealed no abnormalities. Two weeks (**9 weeks of age**) before our consultation, the infant developed respiratory distress characterized by tachypnea (70 breaths/minute), intercostal retractions, and oxygen dependency (FiO_2_ 30%), resulting in a 12‐day (12 weeks of age) admission to the intensive care unit (ICU), as noted in the referral records. The BCG vaccine had not been administered prior to admission, as it is contraindicated in the presence of active TB disease. This clinical decision is correct and crucial. Administering a live bacterial vaccine (BCG) to an infant with active TB is not recommended. The priority is to diagnose and treat the active infection first. According to global and Congolese guidelines, the BCG vaccine can be considered after the completion of successful anti‐TB treatment, provided the child is well and immunocompetent.

During our evaluation, the infant appeared mildly pale with no signs of jaundice. Upon admission to our center, the infant weighed 1700 g, measured 41 cm in length, and had a head circumference of 28 cm and a brachial circumference of 10 cm. Systemic manifestations included poor feeding and intermittent fever (38.5°C). Given the infant’s prolonged clinical history and the mother’s confirmed TB status, currently on antitubercular therapy (ATT), we considered congenital or transmitted TB as a potential diagnosis. The HIV test was negative.

The diagnosis of congenital TB was evaluated using Cantwell’s established criteria, which require two components for a definitive diagnosis:

1. Proven tuberculous lesions in the infant and
2. At least one of the following:
  • **Lesions in the first week of life**, or
  • **A proven tuberculous infection or disease in the mother,** or
  • **Exclusion of the possibility of postnatal transmission** through a thorough contact investigation, or
  • Maternal coma being a novel risk factor.

While genotypic matching of maternal and infant isolates provides the strongest evidence, it is not a mandatory component of the criteria and is often unavailable in resource‐limited settings.

### 1.4. Diagnostic Workup

(1) **Imaging**: Chest X‐ray was normal; lymph node ultrasound showed hypoechoic masses with central necrosis. (2) **Histopathology**: Excisional biopsy of a cervical node revealed caseating granulomas with Langhans giant cells. (3) **Microbiology**: GeneXpert MTB from lymph node aspirate was positive for *Mycobacterium tuberculosis*; lymph node culture could not be performed at our center due to the lack of appropriate culture media. This test, which requires referral to a private laboratory, was not pursued due to financial constraints; blood/CSF cultures were negative for bacterial pathogens. (4) **Maternal–neonatal link**: Genotypic strain analysis (not routinely available in DRC) was not performed, but temporal and clinical evidence strongly supported vertical transmission.

Key diagnostic clues for congenital TB included the following: (1) **Maternal History**: undiagnosed TB (postpartum confirmation) and prolonged maternal coma (suggesting disseminated TB). (2) Histopathology: caseating granulomas with Langhans giant cells in lymph node biopsy. (3) **Microbiology**: positive TB‐PCR (Xpert MTB) or culture from lymph node aspirate. This result came out three weeks after admission in our center (**15 weeks of age = almost 4 months after birth**). (4) **Imaging**: necrotic lymph nodes on ultrasound. Note: The ultrasound and histopathology images are not included in this document because our center is secured with a system that does not allow the downloading of patients’ data.

### 1.5. Treatment and Outcome

ATT was initiated at 4 months of life with rifampicin (10 mg/kg/day), isoniazid (10 mg/kg/day), and pyrazinamide (30 mg/kg/day), **adjusted for prematurity** (isoniazid 8.3 mg/kg, rifampicin 10 mg/kg, and pyrazinamide 25 mg/kg.)

### 1.6. Dosing Decisions and Regimen

ATT was initiated with a daily regimen of isoniazid (H), rifampicin (R), and pyrazinamide (Z), adjusted for the infant’s weight. Daily administration with child‐friendly fixed‐dose combination (FDC) tablets is the WHO‐recommended standard to maximize efficacy and minimize the risk of acquired drug resistance. Based on the infant’s weight of 1.7 kg at the start of ATT, dosing was determined using the WHO weight band for 3 to < 4 kg.

### 1.7. Monitoring Schedule

Clinical and weight‐based monitoring was conducted in alignment with WHO guidelines for HIV‐negative children. The minimum recommended follow‐up assessments for clinical response and adverse effects were scheduled at

• 2 and 4 weeks after treatment initiation.
• At the end of the 2‐month intensive phase.
• Every 2 months thereafter until treatment completion.

Dosages were recalculated with each significant weight gain. Given the infant’s prematurity, monitoring included serial liver function tests (no hepatotoxicity, a heightened concern in this population), weekly weight gain, and gradual resolution of lymphadenopathy over 8 weeks of treatment.

Ultrasound measures, and any abdominal imaging (liver/spleen), representative laboratory ranges, and the infant’s oxygen saturations and heart rate at key points (presentation and ICU transfer) were not available because our center is secured with a system that does not allow the downloading of patients’ records. Maternal follow‐up confirmed the mother received first‐line ATT under directly observed therapy (DOT), with symptom improvement. Neonatal outcome: At 3 months of treatment (7 months of age), the infant showed significant clinical improvement (weight gain and resolved respiratory distress) and continued ATT for a planned 6‐month course (10 months of age).

## 2. Rationale for a Three‐Drug Regimen (HRZ) [8]

Ethambutol (E) was omitted from the intensive phase, resulting in a 2HRZ/4HR regimen. This decision is consistent with WHO recommendations, which state that for children with drug‐susceptible TB, “ethambutol can be omitted for patients who are HIV‐negative or in settings with a low prevalence of HIV or isoniazid resistance.” The infant was confirmed HIV‐negative, and the clinical presentation (nondisseminated lymphadenitis) was not severe, supporting the use of the three‐drug combination.

### 2.1. Regimen Duration Justification

The patient was planned for a total 6‐month course of ATT. For infants under 3 months of age, a standard 6‐month regimen remains the definitive WHO‐recommended course. This aligns with the historical standard 2HRZ(E)/4HR regimen. It is noteworthy that newer 2024 ATS/CDC/ERS/IDSA guidelines recommend a 4‐month regimen (2HRZE/2HR) for children aged 3 months to 16 years with nonsevere TB; however, these international guidelines were not yet standard at the time of this case, and the patient’s age at treatment initiation (4 months) and prematurity warranted a cautious, full‐duration approach.

### 2.2. Patient Perspective

We noted the caregiver’s (paternal aunt of the child) experience, concerns, and hope during the diagnostic journey:

1. Fear and confusion: “Watching our newborn struggle for months without answers was terrifying. We felt lost, moving from clinic to clinic while he grew weaker,” she said.
2. Diagnostic delay: “Our greatest fear was the unknown. For weeks, we saw our son suffer through treatments that didn’t work, not knowing the true enemy was TB,” she continued.
3. Relief after diagnosis: “After months of uncertainty and fear, finally hearing the diagnosis of TB brought a painful clarity, but also the first hope for targeted treatment,” she concluded.

### 2.3. Discussion

This case of congenital tuberculous lymphadenitis in a preterm neonate born to a comatose mother in the DRC underscores critical challenges in diagnosing and managing congenital TB in resource‐limited settings. We were unable to identify prior case reports describing congenital TB in the context of maternal coma to delayed TB diagnosis and subsequent vertical transmission in a preterm infant, providing unique insights into the intersection of maternal critical illness and neonatal TB epidemiology. Pathophysiology and vertical transmission: Congenital TB typically arises from hematogenous spread via the umbilical vein or aspiration of infected amniotic fluid [1]. In this case, maternal coma likely masked early TB symptoms, allowing disseminated disease with bacillemia and transplacental transmission. Maternal coma possibly due to TB meningitis is a hypothesis. The preterm neonate’s immature immune system and lack of passive maternal antibodies (due to untreated TB) further facilitated infection progression [6]. While lymphadenitis is uncommon in congenital TB (reported in only 15%–20% of cases), its presence here suggests hematogenous seeding of lymphoid tissue, compounded by delayed neonatal immune responses [4]. Diagnostic challenges in preterm neonates: Congenital TB is often misdiagnosed as bacterial sepsis or pneumonia due to overlapping symptoms (e.g., respiratory distress and fever) [9]. In this neonate, cervical lymphadenopathy, a rare primary manifestation, initially diverted suspicion toward bacterial or congenital viral infections. Diagnostic delays were exacerbated by limited access to rapid molecular testing (e.g., TB‐PCR), which became available only after referral to a tertiary center. This aligns with studies from sub‐Saharan Africa, where > 60% of congenital TB cases are diagnosed postmortem [10]. Maternal coma as a risk amplifier: Maternal coma during pregnancy represents a critical gap in TB screening protocols. Comatose patients in high‐burden regions are rarely tested for TB unless overt symptoms exist, despite evidence that critical illness increases TB reactivation risk [10]. In this case, the mother’s coma likely reflected advanced TB dissemination, possible meningitis though confirmatory neuroimaging was unavailable, but miliary TB was confirmed by the chest X‐ray. This highlights the urgent need to integrate TB testing into antenatal care for critically ill pregnant women, even in nonrespiratory presentations. Management complications in preterm neonates: Treating congenital TB in preterm infants requires careful ATT dose adjustments due to immature hepatic and renal function [6]. While the WHO recommends standard ATT regimens for neonates, prematurity necessitates vigilant toxicity monitoring, particularly for rifampicin‐induced jaundice and pyrazinamide hepatotoxicity [9]. This infant’s favorable response to weight‐adjusted ATT underscores the feasibility of treatment in resource‐limited settings when initiated early.

Applying the Cantwell criteria, this case meets the definitive diagnosis for congenital TB. First, the infant has proven tuberculous lesions, demonstrated by histopathology (caseating granulomas with Langhans giant cells), and a positive microbiological test (GeneXpert MTB) from a lymph node aspirate. Second, there is definitive evidence of TB disease in the mother, who was diagnosed with AFB smear‐positive pulmonary TB in the immediate postpartum period. The infant’s presentation with lymphadenopathy and subsequent systemic illness in the first few weeks of life, in the absence of BCG vaccination or other documented exposure, fulfills the criterion for vertical transmission. Therefore, despite the lack of genotypic strain comparison, the case satisfies both required components of the Cantwell criteria.

Public health implications: The DRC’s high TB burden (incidence: 322/100,000) and fragmented healthcare system amplify risks of vertical transmission [11].

## 3. Learning Points

This case advocates for the following: (1) **Routine antenatal TB screening**: including GeneXpert MTB testing in comatose or critically ill pregnant women; (2) **Neonatal TB vigilance**: prioritizing TB in preterm neonates with unexplained lymphadenopathy or sepsis‐like illness; and (3**) Maternal DOT integration**: ensuring postpartum maternal TB treatment to reduce postnatal transmission risks.

## 4. Strengths and Limitations

This report provides novel insights into TB transmission dynamics in pregnancies complicated by maternal coma. However, genotypic confirmation of maternal–neonatal TB strain matching was unavailable, a common limitation in high‐burden regions. Additionally, the lack of autopsy or neuroimaging for the mother precluded definitive determination of her coma’s etiology.

### 4.1. Recommendations

Programmatic guidelines in high‐burden, resource‐limited settings should identify critically ill pregnant women as a priority group for TB screening. Recommended actions should be realistically scaled to local capacity. A positive symptom screen must prompt a rapid diagnostic cascade utilizing the most advanced tools accessible on‐site—such as chest X‐ray, where feasible, or molecular testing, where available —to guide timely treatment and mitigate missed diagnoses and poor outcomes.

### 4.2. Conclusion

This congenital TB case in a preterm neonate born to a comatose mother in the DRC highlights the critical risk of vertical transmission due to delayed maternal TB diagnosis amidst severe antenatal illness. It underscores that congenital TB must be suspected in preterm infants with lymphadenopathy born to critically ill mothers in endemic areas, even without classic TB symptoms. Maternal coma represents a high‐risk scenario demanding proactive antenatal TB screening for all critically ill pregnant women, regardless of presentation. Early neonatal diagnosis and weight‐adjusted ATT initiation are feasible and vital for reducing mortality, reinforcing the urgent need to integrate TB screening into antenatal care protocols for critically ill women in high‐burden settings.

**Nomenclature**
AFB: Acid‐fast bacilli
ATT: Antitubercular treatment
DOT: Directly observed therapy
DRC: Democratic Republic of Congo
MTB‐PCR: Mycobacterium tuberculosis polymerase chain reaction
TB: Tuberculosis
WHO: World Health Organization

## Ethics Statement

The authors declare that the research presented in this manuscript adheres to the ethical principles outlined by ethical committee of Goma University. All procedures involving human participants were conducted in accordance with the ethical standards of the Democratic Republic of Congo and the Declaration of Helsinki (1964), as revised in 2013.

## Consent

Written informed consent was obtained from the patient for the publication of this case report and any accompanying images. A copy of the written consent can be obtained on request by the editor‐in‐chief of this journal.

## Disclosure

A preprint has previously been published on the research square preprint server (Kikuhe et al. [12]). All authors read and approved the final manuscript.

## Conflicts of Interest

The authors declare no conflicts of interest.

## Author Contributions

(a) Jason Nzanzu Kikuhe diagnosed the case and managed the patient in collaboration with pediatricians. He also collected the data and wrote the initial draft of the manuscript.

(b) Eugnénie Mbindule Kalere administered treatment and contributed to the drafting and revising of the manuscript.

(c) Castram Mbusa Makata followed‐up the patient in ICU and contributed to the drafting and revising of the manuscript.

(d) Josias Kasereka Songya critically analyzed the text and images and contributed significantly in shaping the final manuscript.

## Funding

This study received no financial support.

## Data Availability

The data that support the findings of this study are available upon request from the corresponding author. The data are not publicly available due to privacy or ethical restrictions.
